# Effects of Human Endothelial Progenitor Cell and Its Conditioned Medium on Oocyte Development and Subsequent Embryo Development

**DOI:** 10.3390/ijms21217983

**Published:** 2020-10-27

**Authors:** Seok Hee Lee

**Affiliations:** 1 Center for Reproductive Sciences, Department of Obstetrics and Gynecology, University of California San Francisco, San Francisco, CA 94143, USA; seokhee.lee@ucsf.edu; Tel.: +1-4154760932; 2 Department of Theriogenology and Biotechnology, College of Veterinary Medicine, Seoul National University, Seoul 08826, Korea

**Keywords:** conditioned medium, enzyme-linked immunosorbent assay, growth factors, human endothelial progenitor cells, oocyte development

## Abstract

Human endothelial progenitor cells (EPCs) secrete numerous growth factors, and they have been applied to regenerative medicine for their roles in angiogenesis as well as neovascularization. Angiogenesis is one of the essential factors for the maturation of ovarian follicles; however, the physiological function of EPCs or their derivatives on in vitro culture systems has not been fully understood. The aim of this study was to evaluate the effectiveness of EPCs and their conditioned medium (EPC-CM) on oocyte development and subsequent embryo development. In the results, the oocyte development and subsequent embryo development were significantly improved in EPCs and the EPC-CM group. In addition, markedly increased levels of growth factors/cytokines, such as basic fibroblast growth factor (bFGF), vascular endothelial growth factor (VEGF), insulin growth factor-1 (IGF-1), interleukin-10 (IL-10), and epidermal growth factor (EGF), were observed in medium from the EPC-CM group. Additionally, EPC-CM after in vitro maturation (IVM) had significantly decreased reactive oxygen species (ROS) levels compared to those of other groups. Transcriptional levels of growth factor receptor-related genes (*FGFR2*, *IGF1R*) and anti-apoptotic-related gene (*BCL2*) were significantly upregulated in cumulus cells/oocytes from the EPC-CM group compared with those from the control. Furthermore, the expression levels of cumulus expansion-related genes (*PTGS2*, *TNFAIP6*, *HAS2*) and oocyte-maturation-related factors (*GDF9*, *BMP15*) were significantly enhanced in the EPC-CM group. Consequently, the present study provides the first evidence that EPC-CM contains several essential growth factors for oocyte development by regulating genes involved in oocyte maturation.

## 1. Introduction

The in vitro-assisted reproduction techniques have been considered an essential tool for studying oocyte maturation, embryo development, and animal model research [1,2]. It has been demonstrated that the intrinsic high quality of oocyte during maturation is a prerequisite condition for supporting the efficiency of preimplantation embryo development as well as fetal growth [3]. Therefore, recently, many studies have been performed to increase the rate of in vitro maturation (IVM) in attempts to better simulate the in vivo microenvironment during IVM. A wide variety of oocyte maturation research has shown the addition of exogenous growth factors in culture media [4,5] and application of a cell-based co-culture system that secretes various kinds of growth factors [6,7,8,9,10,11]. However, as it can be hypothesized that because nutritional complexes, including several cytokines or growth factors, are required for oocyte maturation, supplementing all these growth factors is rather high priced. Additionally, the application of a cell-based co-culture system may become cumbersome, which requires the ideal status of co-culture cells without any contamination until applying IVM.

The secreted factors that exist in the medium where the stem cells are cultured are referred to as secretome, microvesicle, or exosome; therefore, the medium is named conditioned medium (CM) [12]. It has been proved that the CM of cultured stem cells provides a variety of beneficial growth factors secreted by those cells [13,14]. Similarly, it was suggested that CM supernatants derived from human umbilical cord cells might be useful in culture media through their amino acids, cytokines, and vitamins, all of which affect serum components [15,16]. Additionally, CM derived from various types of stem cells has been applied in many different types of diseases and been proven to improve healing and treatment [17,18,19]. As supernatant of cultured cells contains a large number of growth factors, including cytokines and bioactive factors [15], it can be speculated that CM can modulate microenvironment IVM and would provide support for oocyte development.

In our previous study, clinical assessment of human endothelial progenitor cells (EPCs) of dogs showed promising results: increased serum concentration levels of vascular endothelial growth factor (VEGF), and interleukin 10 (IL-10) [20]. It was proved that they are potential candidates as an endogenous repair mechanism in vasculogenesis and neovascularization by secreting multiple growth factors [21,22]. Furthermore, it is widely accepted that these vascular activities by secreting factors have a beneficial role in the maturation of ovarian follicles as well as granulosa and cumulus cell function for further growth [23]. Besides, we demonstrated that EPC co-culture markedly enhanced the oocyte and subsequent embryo development by upregulating cumulus-cell expansion (*HAS2*, *PTGS2*, *TNFAIP6*, and *PTX3*) and oocyte maturation-related genes (*GDF9* and *BMP15*) in cumulus-oocyte complexes (COCs), and pluripotency-related genes (*SOX2*, *OCT4*, and *NANOG*) in blastocysts [11]. Additionally, we found that the EPC co-culture medium contains a high concentration level of VEGF, bFGF, and IL-10 [11]. However, the application of a cell-based co-culture system on IVM would be rather complicated, because the cells need to be prepared approximately 2 days before IVM and the co-culture cells are required to maintain the ideal physiological status without any contamination until application on IVM. If co-culture cells did not conform to these criteria, the experiment could not be performed. According to our previous study, although EPC co-culture showed a potential function on oocyte development, the overall process of the co-culture system is somewhat complicated. As an EPC co-culture indirectly influences oocyte maturation through the transwell system, therefore, I hypothesized that the conditioned medium derived from EPC might show similar or improved effects on oocyte development. If so, the disadvantages of EPC co-culture would be overcome, and there will be a possibility to highlight the potential function of EPC-CM as a paradigm to establish a reliable system for ARTs and give a new insight to the field of reproduction. Through these results, we assumed an indirect approach of conditioned medium derived from EPCs (EPC-CM) might have an effect on oocyte development by paracrine effects. However, still, little information is available on the serial process of EPC-CM during IVM and whether the growth factors/cytokine secreted by EPCs would effectively perform their roles in COCs development.

**Figure 1:**
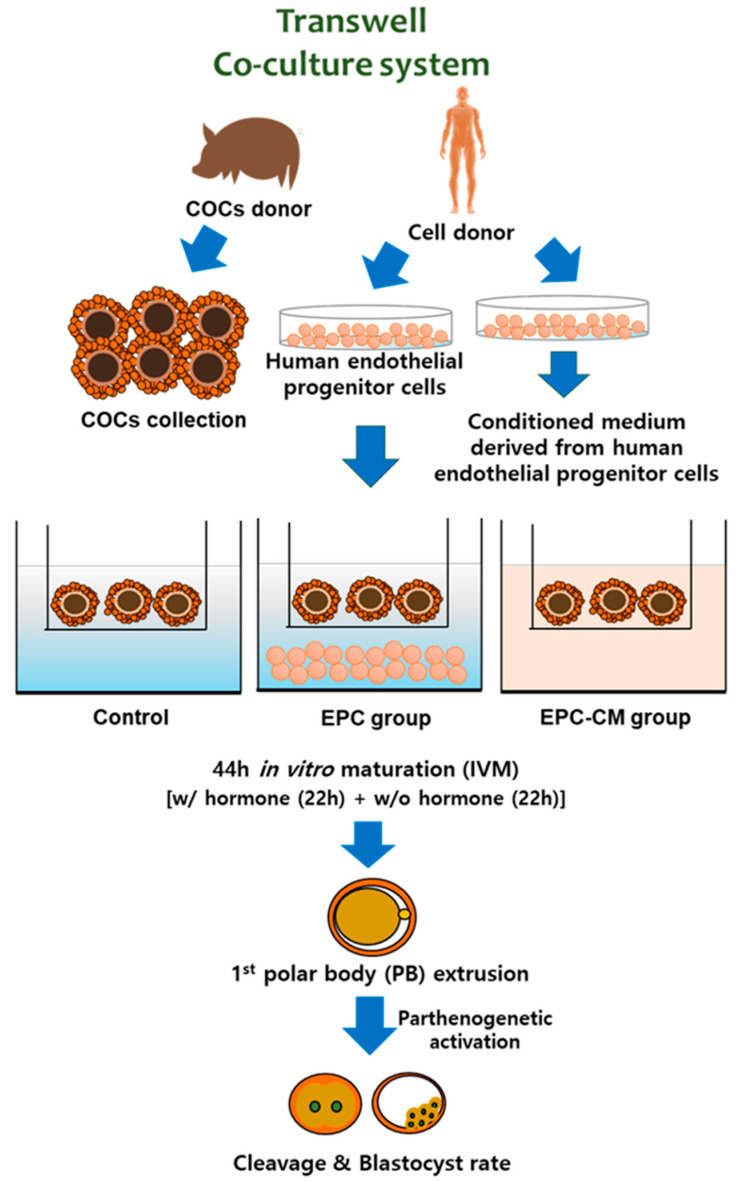
A schematic illustration of the experiments. Co-culture endothelial progenitor cells (EPC) or endothelial progenitor cells-derived conditioned medium (EPC-CM) was applied into a porcine in vitro maturation (IVM) system. After 44 h or in vitro maturation, 1st polar body extrusion from oocytes and subsequent cleavage and blastocyst rate were evaluated. EPC; endothelial progenitor cells, EPC-CM; endothelial progenitor cell-derived conditioned medium.

Thus, we hypothesized that the indirect approach of EPC culture media, which was discarded otherwise as spent media during co-culture, would be an excellent source of growth factors to improve oocyte quality by paracrine effects. To the best of the author’s knowledge, no study has reported on the potential effects of EPC-CM on oocyte development. Therefore, the aim of the study was to evaluate the effect of EPC-CM on porcine oocyte development and subsequent embryo development in vitro in view of the potential effects of secreted factors from EPC as follows: (1) evaluation of the cumulus cell expansion degree and in vitro maturation rate of porcine oocytes in EPC co-culture and EPC-CM groups; (2) quantification of basic fibroblast growth factor (bFGF), VEGF, insulin growth factor-1 (IGF-1), IL-10, and epidermal growth factor (EGF) from culture medium in each group by ELISA analysis; (3) concentration of reactive oxygen species (ROS) from supernatant in each group; (4) subsequent embryo development after parthenogenetic activation (PA); and (5) relative gene expression of growth factors and their receptors in cumulus cells and oocytes. A schematic illustration of the experiment in this study is provided in Figure 1. The present study therefore provides new insight into how EPC-CM affects IVM of porcine oocytes, and provides a strong basis for the procurement of high-quality oocytes for animal reproduction as well as for establishment of a reliable system of assisted reproductive techniques.

## 2. Results

### 2.1. Effect of EPC on Porcine Oocyte Nuclear Maturation and Cumulus Expansion

**Figure 2:**
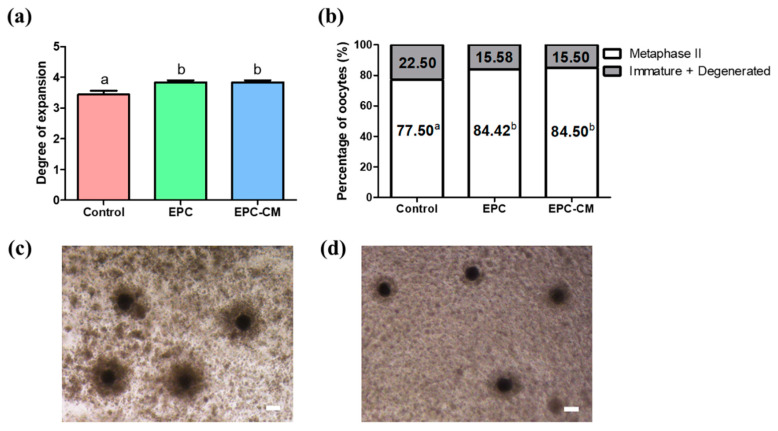
Effect of endothelial progenitor cells/endothelial progenitor cells-derived conditioned medium (EPC/EPC-CM) on oocyte development during in vitro maturation (IVM). (**a**) Degree of expansion in cumulus cells derived from three groups. (**b**) Percentage of matured oocytes or immature/degenerated oocytes derived from three groups. (**c**) Expansion in cumulus cells from the control group. (**d**) Expansion in cumulus cells from the EPC-CM group. A total 690 cumulus-oocyte complexes were used (control: 230, EPC: 230, EPC-CM: 230) and at least 6 biological replications were performed. ^a,b^ Within a column, values with different superscripts are significantly different (*p* < 0.05). Bar represents 100 μm. EPC; endothelial progenitor cells, EPC-CM; endothelial progenitor cell-derived conditioned medium.

In our previous research, we confirmed that EPC maintained cobblestone morphology with proliferation potential during culture [20]. Additionally, every passage of EPC showed a homogeneous population of cells with high expression of CD105, CD31, and CD144 as endothelial progenitor markers [11]. All the cells were positive for Dil-Ac-LDL uptake, which indicated they maintained their endothelial cell function as previously described [20]. Through tube formation assay, EPCs showed their capacity for vasculogenesis, which is believed to be essential in new vessel formation with tube-like structures by fluorescence microscopy. The effects of EPC co-culture and EPC-CM on cumulus expansion of COCs and nuclear maturation of oocytes were investigated. As shown in Figure 2a, the EPC co-culture and EPC-CM groups significantly increased the proportion of COCs exhibiting complete cumulus expansion compared with the control. In addition, we analyzed the effects of EPC on the nuclear maturation of porcine oocytes. As shown in Figure 2b, the rates of oocyte nuclear maturation in the EPC co-culture and EPC-CM group (84.4 ± 0.7% and 84.5 ± 1.4%, respectively) were significantly higher than those in the control group (77.5 ± 1.1%, *p* < 0.05). A total of 690 COCs were used (control: 230, EPC: 230, EPC-CM: 230) and at least 6 biological replications were performed.

### 2.2. Quantification of Secreted Factors Derived from Culture Media

**Figure 3:**
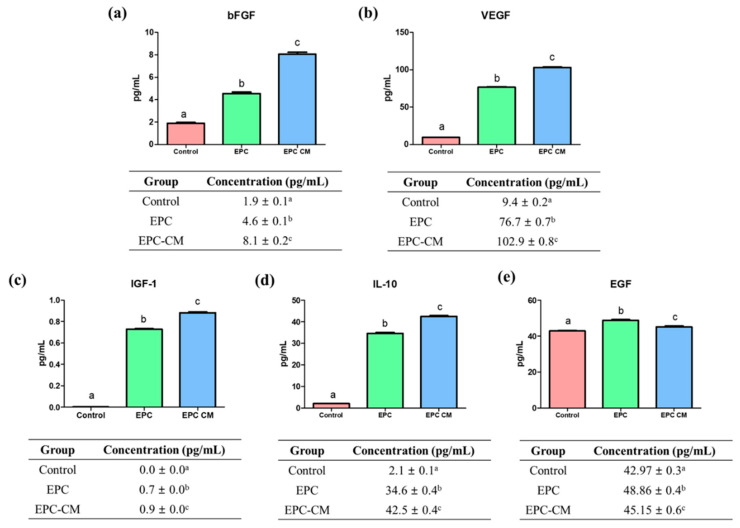
Concentration of (**a**) bFGF, (**b**) VEGF, (**c**) IGF-1, (**d**) IL-10, and (**e**) EGF from supernatant in each group. ^a,b,c^ Within groups, values with different superscript letters are significantly different (*p*  < 0.05). Data are shown as the means ± SEM. An equal volume of supernatant from each group was used and at least six biological replications were performed. bFGF; basic fibroblast growth factor, VEGF; vascular endothelial growth factor, IGF-1; insulin growth factor-1, IL-10; interleukin 10, EGF; epidermal growth factor.

The concentrations of bFGF, VEGF, IGF-1, IL-10, and EGF from culture media in three groups were analyzed (Figure 3). The concentration of bFGF was significantly higher in the EPC-CM group (8.1 ± 0.2 pg/mL, *p* < 0.05) compared with the control and EPC group (control: 1.9 ± 0.1 pg/mL, EPC group: 4.6 ± 0.1 pg/mL, *p* < 0.05). Regarding VEGF concentration, there was significantly the highest level of VEGF in the EPC-CM group (102.9 ± 0.8 pg/mL, *p* < 0.05) compared with the control and EPC group (control: 9.4 ± 0.2 pg/mL, EPC groups: 76.7 ± 0.7 pg/mL) (*p* < 0.05). In case of IGF-1 concentration, the EPC-CM group showed a significantly high level of IGF-1 (0.9 ± 0.0 pg/mL, *p* < 0.05) compared with other groups (control: 0.0 ± 0.0 pg/mL, EPC groups: 0.7 ± 0.0 pg/mL) (*p* < 0.05). Additionally, the highest level of IL-10 was observed in the EPC-CM group (42.5 ± 0.4 pg/mL, *p* < 0.05). Lastly, the levels of EGF were significantly higher in the EPC (48.9 ± 0.4 pg/mL) and EPC-CM group (45.2 ± 0.6 pg/mL) compared with the control (43.0 ± 0.3 pg/mL) (*p* < 0.05). Thus, the levels of bFGF, VEGF, IGF-1, IL-10, and EGF from culture media were significantly increased in the EPC and EPC-CM group compared with the control. Furthermore, the EPC-CM group showed a comparatively higher concentration of bFGF, VEGF, IGF-1, and IL-10 than the EPC group. An equal volume of supernatant from each group was used and at least six biological replications were performed in this experiment.

### 2.3. Quantification of ROS Concentration Derived from Culture Media

**Figure 4:**
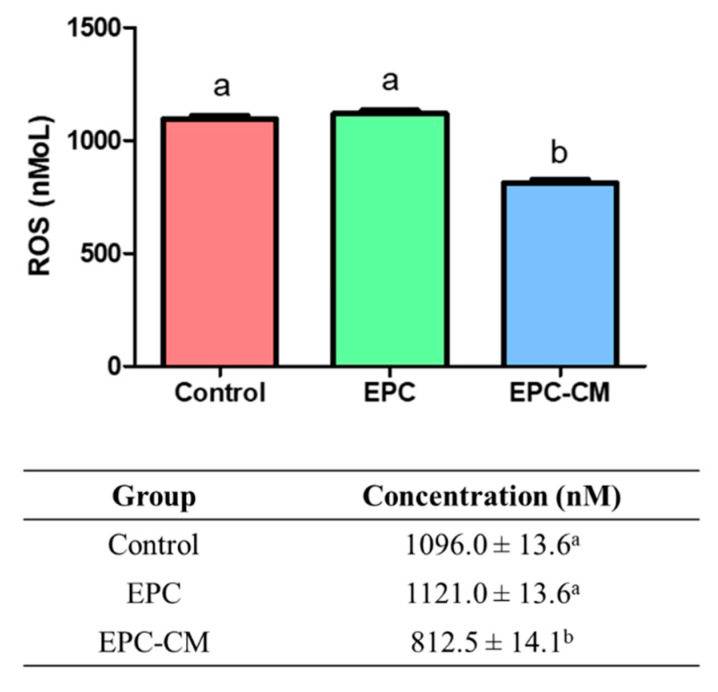
Evaluation of the reactive oxygen species (ROS) concentration from culture medium supernatant in comparison to the control group. ROS from supernatant was measured in three groups. Control; the group without endothelial progenitor cells (EPC) co-culture or endothelial progenitor cells-derived conditioned medium (EPC-CM). EPC; the group co-cultured with EPC, EPC-CM; the group cultured with EPC-CM. The equal volume of supernatant from each group was used and at least six biological replications were performed in this experiment. ^a,b^ Within a column, values with different superscripts are significantly different (*p* < 0.05).

The potential of treatments to induce oxidative stress was evaluated by measuring ROS levels in supernatant culture medium from each group (Figure 4). The production of ROS was significantly lower in the supernatant culture medium derived from the EPC-CM group (812.5 ± 14.1 nM, *p* < 0.05) compared with that of the control and EPC group (control: 1096.0 ± 13.6 nM, EPC group: 1121.0 ± 13.6 nM, *p* < 0.05). An equal volume of supernatant from each group was used and at least six biological replications were performed in this experiment.

### 2.4. Effects of EPC Co-Culture and EPC-CM during IVM on In Vitro Development of Parthenotes

**Figure 5:**
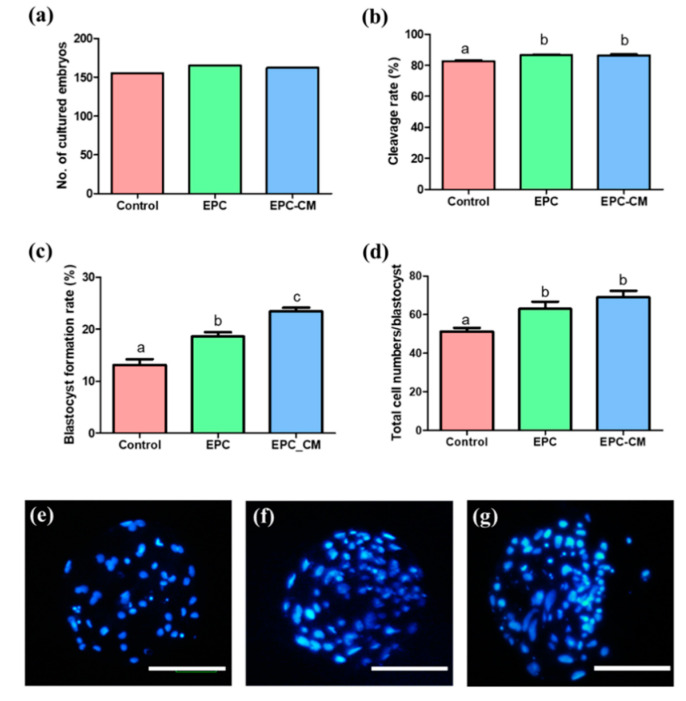
Effect of endothelial progenitor cells/endothelial progenitor cells-derived conditioned medium (EPC/EPC-CM) on subsequent embryo development after oocyte parthenogenetic activation. (**a**) Total number of cultured embryos, (**b**) Cleavage rate, (**c**) blastocyst formation rate, (**d**) total cell numbers in blastocysts derived from each group. (**e**–**g**) Hoechst 33342 staining of porcine embryos at the blastocyst stage using in vitro matured oocytes. (**e**) Blastocyst derived from the control group, (**f**) Blastocyst derived from the EPC group, (**g**) Blastocyst derived from the EPC-CM group. Bar represents 100 μm. Data are shown as the means ± SEM. A total of 566 embryos were used (control: 178, EPC: 194, EPC-CM: 194) and at least six biological replications were performed in this experiment. The blastocyst rate was calculated based on the total embryos in culture. ^a,b,c^ Within a column, values with different superscript letters are significantly different (*p* < 0.05).

As shown in Figure 5, after PA, the cleavage rate of haploid parthenotes derived from the EPC and EPC-CM groups (86.5 ± 0.5 and 86.4 ± 0.7%, respectively, *p* < 0.05) was significantly higher than that of the control (82.6 ± 0.6%, *p* < 0.05). In addition, blastocyst formation in the EPC and EPC-CM groups (18.6 ± 0.8 and 23.4 ± 0.7%, respectively, *p* < 0.05) was higher than in the control (13.1 ± 1.1%, *p* < 0.05), and the EPC-CM group showed a significantly higher blastocyst formation rate compared with the EPC group. With respect to the total cell numbers of blastocysts (Figure 5e–g), the EPC and EPC-CM groups (63.0 ± 3.8 and 69.0 ± 3.3%, respectively, *p* < 0.05) showed a significant increase compared to the control (51.3 ± 1.8, *p* < 0.05). A total of 566 embryos were used (control: 178, EPC: 194, EPC-CM: 194) and at least 6 biological replications were performed in this experiment.

### 2.5. Effects of EPC Co-Culture and EPC-CM during IVM on the Relative Expression of Genes in Cumulus Cells

**Figure 6:**
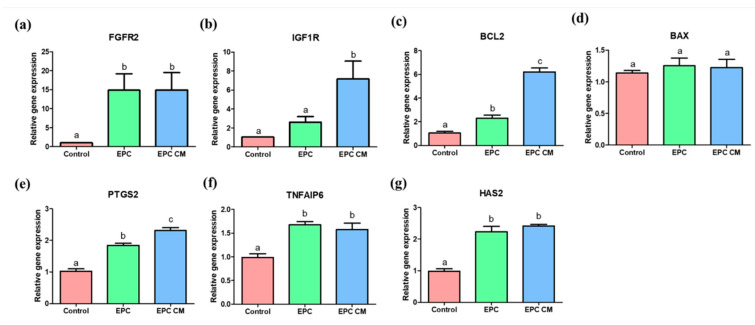
mRNA expression levels of growth factor receptor-related genes (*FGFR2* and *IGF1R*) (**a**,**b**), apoptosis-related genes (*BCL2* and *BAX*) (**c**,**d**), and cumulus expansion-related genes (*PTGS2*, *TNFAIP6*, and *HAS2*) (**e–g**) in cumulus cells. Control: cumulus cells cultured without endothelial progenitor cells (EPC) or endothelial progenitor cells-derived conditioned medium (EPC-CM) during in vitro maturation (IVM). EPC; cumulus cells co-cultured with EPC during IVM. EPC-CM: cumulus cells cultured with EPC-derived conditioned medium. Data are shown as means ± SEM. ^a,b,c^ Within a column, values with different superscript letters are significantly different (*p* < 0.05). A total of 85 randomly selected cumulus-oocyte complexes (COCs) derived from each group were used and at least nine technical replications were performed in this experiment. *FGFR2*; fibroblast growth factor receptor 2, *IGF1R*; insulin growth factor 1 receptor, *PTGS2*; prostaglandin-endoperoxide synthase 2, *TNFAIP6*; tumor necrosis factor α-induced protein 6, *HAS2*; hyaluronan synthase 2.

The relative expression of genes related to oocyte maturation and apoptosis was analyzed in porcine cumulus cells derived from COCs after IVM. The expression of fibroblast growth factor receptor 2 (FGFR2) was significantly increased in the cumulus cells derived from the EPC and EPC-CM groups compared with the control (Figure 6a). The cumulus cells derived from the EPC-CM group showed significantly increased gene expression of insulin growth factor 1 receptor (IGF1R) compared with those of the control and EPC group (Figure 6b). As for apoptosis gene expression, there was significantly increased expression of BCL2 in cumulus cells derived from the EPC and the EPC-CM groups (Figure 6c) and the EPC-CM group showed a significantly increased level of BCL2 compared to the EPC group; however, no significant difference in expression of BAX was observed among all groups (Figure 6d). Additionally, the cumulus expansion-related genes, such as PTGS2, TNFAIP6, and HAS2, were significantly upregulated in cumulus cells from the EPC and EPC-CM groups (Figure 6e–g). In particular, the expression level of PTGS2 was markedly increased in cumulus cells in EPC-CM compared to those from the EPC group (Figure 6e). A total of 85 randomly selected COCs derived from each group were used and at least 9 technical replications were performed in this experiment.

### 2.6. Effects of EPC Co-Culture and EPC-CM during IVM on the Relative Expression of Genes in Oocytes

**Figure 7:**
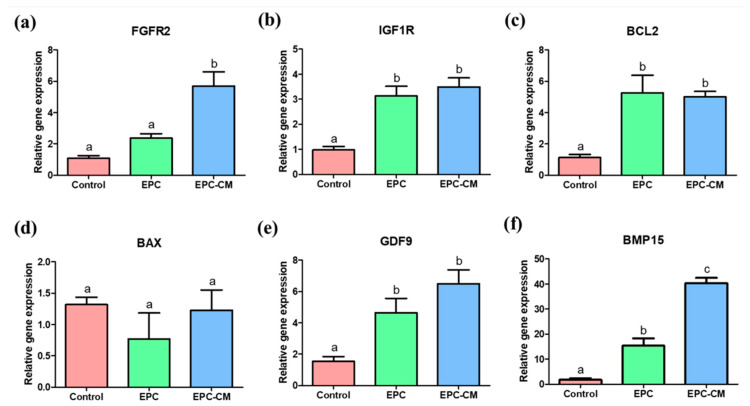
mRNA expression levels of growth factor receptor-related genes (*FGFR2* and *IGF1R*) (**a**,**b**), apoptosis-related genes (*BCL2* and *BAX*) (**c**,**d**), and oocyte maturation-related genes (*GDF9* and *BMP15)* (**e**,**f**) in oocytes. Control: oocytes cultured without endothelial progenitor cells (EPC) or endothelial progenitor cells-derived conditioned medium (EPC-CM) during in vitro maturation (IVM). EPC; oocytes co-cultured with EPC during IVM. EPC-CM: oocytes cultured with EPC-derived conditioned medium. Data are shown as means ± SEM. ^a,b,c^ Within a column, values with different superscript letters are significantly different (*p* < 0.05). A total of 85 randomly selected cumulus-oocyte complexes (COCs) derived from each group were used and at least nine technical replications were performed in this experiment. *FGFR2*; fibroblast growth factor receptor 2, *IGF1R*; insulin growth factor 1 receptor, *GDF9*; growth differentiation factor 9, *BMP15*; bone morphogenetic protein 15.

The relative expression of genes related to oocyte maturation and apoptosis was analyzed in porcine oocytes derived from COCs after IVM. The expression of FGFR2 was significantly upregulated in the oocytes derived from the EPC-CM group compared with the other two groups (Figure 7a). In addition, the expression of IGF1R and GDF9 was significantly increased in the EPC and EPC-CM group compared with the control (Figure 7b,e). Furthermore, the oocytes derived from the EPC and EPC-CM groups showed significantly increased gene expression of BMP15 compared with the control, and the EPC-CM group showed significantly higher expression of BMP15 than that of the EPC group (Figure 7f). In regard to apoptosis gene expression, there was significantly increased expression of BCL2 in oocytes derived from the EPC and EPC-CM groups (Figure 7c); however, no significant difference in the expression of BAX was observed among all groups (Figure 7d). A total 85 randomly selected COCs derived from each group were used and at least 9 technical replications were performed in this experiment.

## 3. Discussion

To our best knowledge, for the first time, it suggests that the co-culture of EPCs with porcine oocytes or the addition of EPC-CM in culture medium during IVM improved the oocyte maturation rate as well as the blastocyst formation rate after PA (Figure 2 and Figure 5). Additionally, we observed that a large amount of growth factors/cytokines, such as bFGF, VEGF, IGF-1, IL-10, and EGF, exist in EPC-CM, and decreased levels of ROS were detected in EPC-CM (Figure 3 and Figure 4). In addition, the COCs derived from the EPC-CM group enhanced the degree of cumulus cell expansion and gene expression levels related to oocyte development (Figure 2, Figure 6 and Figure 7).

Up to date, many studies have suggested that culture conditions, media composition, and growth factors influence oocyte development during IVM and the subsequent developmental capacity [24,25]. However, still, there is a low efficiency of current commercial IVM conditions for improving the maturation of oocytes and their subsequent embryo development compared with in vivo [26]. The conditions of IVM culture media have an effect on the alteration of mRNA and protein content, and ultimately influence oocyte maturation through cellular and molecular processes [27]. In recent decades, numerous studies have attempted to overcome the absence of the follicular microenvironment during IVM by addition of exogenous growth factor [28], follicular fluid [29], and mimicking the in vivo microenvironment with co-culture systems by using fresh oocytes [30], denuded oocytes [31], and oviduct cells [6,7]. In particular, the co-culture system can create an in vivo microenvironment as closely as possible through the secretion of autocrine and paracrine factors, such as hormones, mRNAs, and growth factors, into the culture medium [8,32]. Therefore, the presence of these factors in the conditioned medium can exert their stimulatory/supportive effect on in vitro oocyte development, which is validated by our findings.

It has been demonstrated stem cells secrete a variety of cytokines and growth factors, such as EGF, IL, LIF, FGF2, TGF-β, and IGF-1, that regulate the physiological conditions of recipient cells [33,34,35,36]. The beneficial effects of these factors on IVM have been demonstrated in several species, such as human [37,38], cow [39], horse [40], pig [41], and mouse [38]. In the present study, it was demonstrated that EPC can secrete various paracrine factors, including bFGF, VEGF, IGF-1, and IL-10, into the extracellular environment, which is consistent with previous findings [42,43,44]. Subsequently, the factors derived from EPC-CM effectively activate the mRNA transcript levels of the receptors (*FGFR2* and *IGF1R*) on COCs during IVM. Collectively, it was validated that EPC-CM maintains suitable levels of the growth factors/cytokine in culture medium during IVM, which positively affect the oocyte competency and subsequent embryo development.

In the process of follicular development and luteinization, angiogenesis has been considered as one of the essential processes [45,46]. As the extensive microvascular network supports the delivery of gonadotropin, follicular development is initiated. Among the numerous soluble angiogenic factors involved in microvascular networks, VEGF plays a key role in endothelial cell proliferation and survival of vascular endothelial cells [47]. Within the follicular environment, VEGF activates follicle growth and its physiological activity [48,49]. Moreover, the multiple factors secreted by EPCs are involved in primordial/primary/secondary follicle growth as well as oocyte survival [50,51,52]. In line with their results, this study demonstrated that a large amount of growth factors/cytokines, such as bFGF, VEGF, IGF-1, EGF, and IL-10, derived from EPC-CM existed in the culture medium during IVM (Figure 3), which indicates that the enriched cytokines and growth factors secreted by EPC play an essential role in oocyte development and subsequent embryo development by upregulating mRNA expression (Figure 2 and Figure 5–7). Additionally, the results indicate EPC-CM provides as good or better support than the cells themselves for oocyte development, which would be more appealing to regulatory bodies for oocyte development as EPC-CM is a non-cellular liquid and excludes the potential possibility of contamination. In addition, it can be assumed that EPC also consumed various growth factors in culture medium during IVM to maintain their physiological characteristic and functions. Therefore, the levels of growth factors in the EPC-CM group were comparably higher than those in the EPC group.

The growth factor receptor signaling genes, such as *FGFR2* and *IGF1R*, were analyzed in cumulus cells and oocytes in this study (Figure 66a,b and Figure 7a,b). It has already been reported that IGF-1 has an essential role in nuclear and cytoplasmic maturation of oocytes, exerting its effect through cumulus/granulosa cells by regulation of cumulus cell proliferation and by suppressing apoptosis [53], or stimulating mitogenesis and steroidogenesis of follicular cells [54]. Other studies suggested that IGF-1 are potent stimulators of both resumption and completion of porcine oocyte nuclear maturation [55,56]. The locally secreted/circulating IGF-1 activates IGF1R or its mRNA level in the porcine ovary [57]. The IGF1R is a member of the tyrosine kinase receptor family and it stimulates both the MAPK1/3 and phosphatidylinositol 3-kinase/AKT pathways, which are essential mechanisms underlying the cooperation with the IGF system in follicular differentiation [58]. In the present study, it was confirmed that there was a significantly higher level of IGF-1 in EPC-CM during IVM (Figure 3c). Therefore, it suggests that the increased level of IGF-1 could significantly upregulate *IGF1R* expression in cumulus cells and oocytes after culture with EPC-CM. Another potential paracrine-acting factor that regulates follicular development and oocyte maturation is the fibroblast growth factors (FGFs). It has been well known that FGFs exert their effects on cell proliferation, morphogenesis, and angiogenesis [59,60]. Additionally, FGFs mediate the communication between theca cells and granulosa/cumulus cells [61] and activate glycolysis in cumulus cells [62]. In particular, the bFGF improves the maturation of COCs by increasing cumulus expansion-related genes in pig [28], and their receptors were expressed in follicular cells in humans [63] and cows [64], which are distinct features in follicle development. Transcripts for the four *FGFR* types were present in cumulus and oocytes, and *FGFR2* was most abundant in bovine oocytes among *FGFR* [65]. In addition, *FGFs* and *FGFR2* mRNA expression in follicular cells is significantly higher than before maturation [66], which indicates *FGF/FGFR2* might be associated with follicular development. Interestingly, a report demonstrated that FGFs positively influenced the expression of *BMP15* in oocyte and cumulus expansion as well as subsequent embryo development [65], which is consistent with our findings. In line with their results, it can be indicated that a significantly high level of bFGF in EPC-CM activates the mRNA transcript levels of *FGFR2*/*PTGS2* in cumulus cells and *FGFR2*/*BMP15* in oocytes during IVM (Figure 3, Figure 6 and Figure 7), which might modulate the cumulus expansion as well as high embryo developmental competence.

IL-10 is an anti-inflammatory cytokine that plays an important role in regulating immune responses [67]. Bone marrow-derived macrophages co-cultured with EPC secreted a higher level of IL-10 than BMDM cultured alone in an in vitro system [68]. It has been demonstrated that IL-10 plays a role in the bi-directional communication between oocytes and granulosa cells in pigs [69], and IL-10 shows strong correlations with follicular-derived VEGF in in vitro fertilization [70]. The observations in this research have shown that the production of IL-10 by EPC in a conditioned medium might have an effect on oocyte development (Figure 3d). Consequently, EPC-CM contributed to enhancing COC matrix production and oocyte nuclear maturation because of the EPC-CM-derived factors, including bFGF, VEGF, IGF-1, and IL-10.

ROS are molecules that are responsible for the deleterious effects of oxidative stress. As ROS are produced during metabolic processes in all living beings, much attention has been paid to the role of ROS, including superoxide and hydrogen peroxide, in IVM of the oocyte [71]. They play a key role as a signaling molecule in folliculogenesis and mediate oocyte maturation, meiotic arrest, and resumption [72]. Increased ROS production induces alteration of the microtubule organization and chromosomal alignment of the metaphase II meiotic spindles in the oocytes in mice [73]. Additionally, excessive ROS levels in oocytes impair developmental competence [74]. Thus, it can be implied that the function of ROS is essential for the development of a competent oocyte. In the present study, the concentration of ROS was significantly reduced in the EPC-CM group compared with other groups (Figure 4). Thus, it can be speculated that the EPC-CM has efficient ROS scavenging activity, which could improve the maturation of oocytes and their subsequent embryo development.

As the ROS levels were decreased in the EPC-CM group, it can be assumed that these levels would be closely associated with the expression of apoptosis-related genes in cumulus cells and oocytes during IVM. Numerous investigations have described that the high expression of *BCL2* is related to good-quality oocytes [75,76]. Embryonic development can be influenced by the functional balance of apoptosis and cellular proliferation. Furthermore, the *BCL2* gene family plays a main role in apoptotic pathways [77]. When maturation is prolonged, the ratio of *BAX* to *BCL2* increases significantly; thus, *BCL2* and *BAX* are key in evaluating oocyte aging [78]. In this study, a significantly increased level of *BCL2* was observed in oocytes from the EPC/EPC-CM group compared with the control (Figure 7c). In particular, cumulus cells derived from EPC-CM showed the highest expression level of *BCL2* (Figure 6c), which suggests that EPC-CM could alleviate apoptotic stress in COCs during IVM.

With respect to the possible indirect action of co-cultured EPCs on porcine oocytes, EPC-CM significantly improved nuclear maturation. The *GDF9* and *BMP15* genes have been described as essential members of oocyte-paracrine factors expressed in oocytes [79,80]. The translated protein levels were closely associated with the mRNA transcript levels of *GDF9* and *BMP15* in porcine COCs during IVM [81]. In addition, the mRNA expression level of *GDF9* and *BMP15* on late maturation time would indicate a higher level of GDF9 and BMP15 protein expression compared to those at the same stage of general IVM [82]. Such molecules act synergistically in the development of COCs by regulating multiple cumulus cell functions, including hyaluronic acid synthesis and cumulus expansion [83], and Su et al. [84] suggested that GDF9 and BMP15 induced an oocyte-granulosa cell regulatory loop, affecting oocyte maturation and cumulus expansion. It has been demonstrated that high mRNA expression of *GDF9* and *BMP15* in matured oocytes activates cumulus cell expansion during IVM by increasing levels of cumulus expansion-related genes, such as *HAS2*, *TNFAIP6,* and *PTGS2* [81,85,86]. In the present study, the mRNA expression levels of *GDF9* and *BMP15* was increased in the EPC and EPC-CM groups (Figure 7e,f). In particular, the *BMP15* levels were significantly increased in the EPC-CM group compared with other groups (Figure 7f). Additionally, cumulus expansion-related genes were significantly increased in the EPC-CM group (Figure 7e,f). Taking into consideration the mRNA expression patterns observed, it can be suggested that conditioned medium derived from EPC induces the high quality of oocytes and subsequent embryo development by regulating gene expression, which involves oocyte development. It can be considered that a large number of different samples are necessary to standardize stem cell-derived conditioned medium to achieve adequate reproducibility, which is one of the biggest challenges in current stem cell research. Although the number of samples used in this study is not enough to standardize, we believe that at least this research can provide the valuable possibility of application as well as suggest a new finding for potential functions of EPC-CM on oocyte development, which would provide a new insight in mammalian reproduction.

According to our knowledge, this is the first study about the impact of EPC culture media conditions on porcine oocyte analyzed by ELISA and real-time PCR. Through this approach, we showed that modifications of IVM medium composition markedly affected oocyte development and subsequent embryo development in a differential manner. Therefore, our results provide new insights regarding the physiology of oocyte maturation and highlight the functional importance of in vitro culture conditions.

## 4. Materials and Methods

### 4.1. Ethical Approval and Statement of Informed Consent

This study was approved by the Life Ethics Committee of the Biostar Stem Cell Technology (RBIO 2015-12-001), and all patients gave informed consent for inclusion in the study and obtaining for human endothelial progenitor cells. All experimental research on humans is in compliance with the Helsinki Declaration.

### 4.2. Chemical

All chemicals were obtained from Sigma-Aldrich Co. LLC. (St. Louis, MO, USA) unless otherwise stated.

### 4.3. Isolation, Culture, and Characterization of Human Endothelial Progenitor Cells (EPCs)

Human peripheral blood samples were obtained from healthy donors from Biostar Stem Cell Technology (RBIO 2015-12-001). Briefly, Ficoll-Hypaque (GE Healthcare Life Science, Piscataway, NJ, USA) density gradient centrifugation (2500 rpm, 30 min) was performed to separate peripheral blood mononuclear cells (PBMCs). The PBMCs were collected from the interface between the plasma layer and the Ficoll-Hypaque layer [32]. The cells were seeded into a fibronectin-coated T25 flasks at 1–3 × 10^7^ cells per flask with Defined Keratinocyte-SFM-based medium containing 0.2 mM ascorbic acid, 10 µg/mL L-glutamine, 10 ng/mL human epidermal growth factor, 5 µg/mL insulin, 1ng/mL selenium, 74 ng/mL hydrocortisone, 5 ng/mL Lin28, 1% antibiotic-antimycotic, and 10% FBS, and incubated at 37 °C under 5% CO_2_ in air. The medium was replaced on day 2 (day 0; the day when EPC were seeded) and changed twice a week. The EPC colonies appeared after 2–4 weeks of incubation. When they reached 70–90% confluence, they were passaged into T25 flasks or 6-well plates in proportion to each colony size. In our previous research, we identified and characterized EPCs based on (1) cell surface markers (CD144, CD31, CD105, CD133, CD45, CD14), (2) positive staining for Dil-acetylated low-density lipoprotein confirming endothelial cell function, and (3) observation of tube formation indicating the capacity for vasculogenesis [11,20].

### 4.4. Preparation of Human Endothelial Progenitor Cell Conditioned Medium (EPC-CM)

For the preparation of EPC conditioned medium (EPC-CM), EPCs were thawed and cultured until they reached about 70–80% confluency in a 12-well plate with Defined Keratinocyte-SFM-based medium at 37 °C under 5% CO_2_ in air. Then, the medium was replaced with serum-free Dulbecco’s Modified Eagle’s Medium (DMEM; Invitrogen, Grand Island, NY, USA). The supernatant was collected after 24 h and centrifuge at 13,000× *g* for 5 min at 4 °C, and then the filtration was performed by using a 0.22-µm filter.

### 4.5. In Vitro Maturation of Oocytes by Co-Culture with Human Endothelial Progenitor Cells (EPCs) and EPC Conditioned Medium (EPC-CM)

Porcine ovaries were obtained from sows at a local slaughterhouse. The ovaries were transported to the laboratory in 0.9% NaCl at 32–35 °C within 3 h. The COCs were collected from 3-6-mm-diameter follicles by using an 18-gauge needle on a 10-mL syringe. The COCs were washed three times in the washing medium containing 9.5 g/L of TCM-199, 2 mM sodium bicarbonate, 10 mM HEPES, 0.3% polyvinyl alcohol, 5 mM sodium hydroxide, and 1% penicillin-streptomycin (Invitrogen). On the basis of the following morphological features, COCs were categorized: three or more compact multilayers of cumulus cells and homogeneous cytoplasm. COCs were cultured in IVM medium containing TCM-199 supplemented with 0.57 mM cysteine, 0.91 mM sodium pyruvate, 5 μL/mL insulin–transferrin–selenium solution 100× (Invitrogen), 10 IU/mL equine chorionic gonadotropin (eCG), and 10 IU/mL human chorionic gonadotropin (hCG) in a culture plate.

The presumptive COCs were randomly divided into three groups (control, EPC co-culture group, and EPC-CM group) and cultured in respective culture media. In order to perform EPC co-culture experiments, EPCs were used when they had reached about 70% confluency in a 12-well plate with the medium. The medium was replaced with an IVM medium when co-culture was performed. The 12-well plates were supported with 1.0-μm Transwell polyester membrane inserts (400 μL media per inserts; Corning Inc., Pittston, PA, USA) to allow mutual communication between porcine oocytes and EPCs for a total of 44 h at 39 °C in a humidified atmosphere of 5% CO_2_ in IVM medium. The transwell system provides oocytes with paracrine growth factors derived from EPC through permeable supports with microporous membranes. The intercellular communication distance was approximately 2 mm. For the EPC-CM group, freshly obtained EPC-CM (combine IVM medium and EPC-CM in the ratio of 1 to 1) were used in a 12-well plate during IVM. The control group was maintained under the same conditions as the EPCs and EPC-CM groups except the COCs were cultured without co-culture cells or CM support. The COCs were cultured for 22 h with 10 IU/mL eCG and 10 IU/mL hCG, then washed twice in hormone-free medium. Subsequently, the COCs were cultured for 22 h in IVM medium without hormones. After 44 h of culture for IVM, the COCs were denuded with 0.1% hyaluronidase by gently pipetting and cumulus cells were separated from COCs by centrifugation (2 min, 1975× *g*) and the samples were immediately stored at −80 °C until being used for further experiments. For assessment of the in vitro maturation rates, extrusion of the first polar body (Metaphase II) was evaluated under the stereomicroscope (TE2000-S; Nikon, Tokyo, Japan) with magnification ×80.

### 4.6. Cumulus Expansion Assessment

The degree of cumulus expansion in oocytes was evaluated after 44 h of IVM. The degree of cumulus expansion was determined by the morphology of COCs. In brief, a degree of 0 represented no expansion, characterized by detachment of cumulus cells from the oocyte to assess a flattened monolayer appearance, suggesting a partially or fully denuded oocyte. In case of degree 1, there was no expansion but compacted cumulus cells remained around the oocyte. A degree of 2, only the outermost layers of cumulus cells had expanded, and degree 3 represented that all cumulus cell layers except the corona radiata (cells most proximal to the oocyte) markedly were expanded. A degree of 4 indicated the maximum degree of expansion including the corona radiata.

### 4.7. ELISA Analysis

The concentrations of bFGF (MyBioSource, San Diego, CA, USA), VEGF (R&D Systems, Minneapolis, MN, USA), IGF-1 (R&D Systems, Minneapolis, MN, USA), IL-10 (R&D Systems, Minneapolis, MN, USA), and EGF (R&D Systems, Minneapolis, MN, USA) in supernatant derived from three groups after IVM were analyzed by enzyme-linked immunosorbent assay (ELISA). The assay was performed following the manufacturer’s instructions. Briefly, standard solutions and samples were added to each well of the ELISA plates and incubated for 1–2 h at room temperature. Wash buffer was applied to each well five times to remove unbound antigen. Then, conjugates were added to each well and incubated for 1–2 h at room temperature and washed five times with wash buffer. The substrate solutions were added to each well and incubated for 30 min at room temperature with protection from light. Lastly, stop solutions were added to each well and gentle tapping was performed to ensure thorough mixing. The spectroscopic absorbance of each well was determined immediately with a microplate reader (Tecan Sunrise, Hayward, CA, USA) at 450 nm excitation/590 nm emission.

### 4.8. Assessment of In Vitro ROS Levels in Media

ROS concentration was measured using an OxiselectTM In vitro ROS/RNS Assay kit (Cell Biolabs, San Diego, CA, USA). The assay was performed following the manufacturer’s instructions. Briefly, the media after IVM were collected from three groups to measure the free radical presence in each sample. All samples were transferred into 1.5-mL tubes and centrifugation was performed at 10,000× *g* for 5 min. Each sample (50 μL) was added to wells of a 96-well plate. Then, 50 μL of catalyst were added to each well and incubated for 5 min at room temperature. Lastly, 100 μL of DCFH solution were added to each well followed by incubation at room temperature for 15 min. The fluorescence intensity was measured using a fluorescence plate reader at 480 nm excitation/530 nm emission (Sunrise, Tecan, Austria). The ROS concentrations were evaluated by comparison with the predetermined DCF standard curve.

### 4.9. Parthenogenetic Activation and In Vitro Culture of Parthenotes

After 44 h of IVM, the COCs were denuded with 0.1% hyaluronidase by gently pipetting and denuded oocytes were washed in TALP medium. The matured oocytes were gradually equilibrated in an activation solution composed of 0.28 M mannitol, 0.5 mM HEPES, 0.1 mM MgSO_4_, and 0.1 mM CaCl_2_. Then, the oocytes were placed between two electrodes filled with activation medium in a chamber connected with a BTX Electrocell Manipulator ECM 2001 (BTX Inc., San Diego, CA, USA). The oocytes were activated using a single direct current pulse of 1.5 kV/cm for 60 μs. The activated oocytes were washed 3 times in serum-free porcine zygote medium-5 (PZM-5; Funakoshi Corporation, Tokyo, Japan), and were transferred into the drops of PZM-5 covered with mineral oil in a 4-well dish. The activated oocytes were cultured for 7 days at 39 °C in a humidified atmosphere of 5% O_2_, 5% CO_2_, and 90% N_2_. The cleavage rate of embryos was assessed on days 2 of in vitro culture (IVC) under a stereomicroscope. The blastocyst development rate of embryos was evaluated on day 7 of IVC, and the rate of blastocysts was calculated based on the total activated oocytes. To count the total cell number in blastocysts, they were stained with 5 μm/mL of Hoechst 33342 for 7 min. Then, the blastocysts were mounted on a glass slide with glycerol drops. The total cell number was assessed under a fluorescence microscope (Nikon Corp., Tokyo, Japan) at × 400 magnification.

### 4.10. Total RNA Extraction and cDNA Synthesis

Total RNA was extracted from cumulus cells and oocytes derived from COCs in each group using the Easy-spinTM (DNA-free) Total RNA Extraction Kit (iNtRON Biotechnology Inc., Kyunggi, Korea) following the manufacturer’s instructions. A total of 85 randomly selected COCs derived from each group were used in this experiment. The total RNA concentration was quantified using spectrophotometry (NanoDrop 2000, Thermo Fisher Scientific Inc, Waltham, MA, USA) and samples were immediately stored at −80 °C until being used for cDNA synthesis. Total RNA was reverse transcribed into cDNA using amfiRivert II cDNA Synthesis Premix (GenDEPOT, Barker, TX, USA) according to the manufacturer’s instructions.

### 4.11. Real-Time PCR

The primers for FGFR2, IGF1R, BCL2, BAX, GDF9, BMP15, PTGS2, HAS2, TNFAIP6, and GAPDH genes were designed from sequences of porcine genes obtained from NCBI; all primer sequences were standardized using a standard curve and are listed in Table 1. The expression of each target gene was quantified relative to that of the internal control gene (*GAPDH*). Real-time PCR was performed using an ABI 7300 Real-Time PCR System (Applied Biosystems, Foster City, CA, USA) according to the manufacturer’s instructions with minor modification. Briefly, the total volume of the PCR reaction mixture was 20 μL, composed of 2 μL cDNA, 200 nM forward primer, 200 nM reverse primer, 10 μL SYBR Green interaction dye (Takara Bio USA Inc., Mountain View, CA, USA), and 7.2 μL nuclease-free water in a real-time PCR plate (MicroAmp optical 96-well reaction plate, Singapore). The final concentration of cDNA in each well was 50 ng/μL. The reactions were carried out for 40 cycles with the following parameters of cycles: (1) denaturation at 95  °C for 30 s, (2) annealing at 55 °C for 30 s, and (3) extension at 72 °C for 30 s. The expression of each target gene was quantified relative to that of the internal control gene (*GAPDH*) using the equation, *R* = 2^−[ΔCt sample − ΔCt control]^.

### 4.12. Statistical Analysis

All data were analyzed by one-way ANOVA followed by Tukey’s multiple comparison test using GraphPad Prism 5.0 (Graphpad, San Diego, CA, USA). Values are means ± standard error of the mean. Probability values less than 0.05 were considered to be statistically significant.

## 5. Conclusions

The present study provides evidence on the effect of combined EPC-CM supplementation of IVM medium on porcine oocyte meiotic competence, and subsequent developmental competence. A large amount of growth factors/cytokine in EPC-CM might provide a beneficial effect for oocyte development. Taken together, the findings from our study indicate that EPC-CM supplementation of IVM medium could be used to improve the current IVM system and ensure a better timing to develop into the blastocyst stage. Additionally, EPC-CM is likely to generate a microenvironment that is more appropriate for inducing oocyte maturation and increasing the development of embryos, which provides a new strategy for studying the potential effects of EPC on future assisted reproductive technology procedures.

## Figures and Tables

**Table 1:** Sequence-specific primers used for quantification of differentially expressed transcripts.

Gene	Primer Sequences (5′ → 3′)	GenBank No.	Product Size (bp)
*GAPDH*	F-CTTCCACTTTTGATGCTGGGG R-TCCAGGGGCTCTTACTCCTT	NM_001206359.1	145
*FGFR2*	F: TCATCTGCCTGGTTGTGGTC R: CGCAGCCACGTAAACTTCTG	NM_001099924.2	140
*IGF1R*	F: CCCAATGGCAACCTGAGCTA R: TCCTCGACATCAATGGTGCC	NM_214172.1	137
*BCL2*	F-AGGGCATTCAGTGACCTGAC R-CGATCCGACTCACCAATACC	NM_214285	193
*BAX*	F-TGCCTCAGGATGCATCTACC R-AAGTAGAAAAGCGCGACCAC	XM_003127290	199
*GDF9*	F-ACATGACTCTTCTGGCAGCC R-ACCCTCAGACAGCCCTCTTT	NM_001001909.1	140
*BMP15*	F-AGCTCTGGAATCACAAGGGG R-ACAAGAAGGCAGTGTCCAGG	NM_001005155.1	123
*PTGS2*	F-TGGGGAGACCATGGTAGAAG R-CTGAATCGAGGCAGTGTTGA	NM_214321.1	142
*HAS2*	F-AGTTTATGGGCAGCCAATGTAGTT R-GCACTTGGACCGAGCTGTGT	AB050389	101
*TNFAIP6*	F-AGAAGCGAAAGATGGGATGCT R-CATTTGGGAAGCCTGGAGATT	NM_001159607	106

All primers showed an efficiency between 90 and 110% and a coefficient value >0.9.

## Abbreviations

EPC	Endothelial progenitor cells
EPC-CM	Endothelial progenitor cells-derived conditioned medium
CMbFGF	Conditioned mediumBasic fibroblast growth factor
VEGF	Vascular endothelial growth factor
IGF-1	Insulin growth factor 1
IL-10	Interleukin 10
EGF	Epidermal growth factor
IVM	In vitro maturation
IVC	In vitro culture
ROS	Reactive oxygen species
FGFR2	Fibroblast growth factor receptor 2
IGF1R	Insulin growth factor 1 receptor
PTGS2	Prostaglandin-endoperoxide synthase 2
TNFAIP6	Tumor necrosis factor α-induced protein 6
HAS2	Hyaluronan synthase 2
GDF9	Growth differentiation factor 9
BMP15	Bone morphogenetic protein 15
ELISA	Enzyme-linked immunosorbent assay
COCs	Cumulus-oocyte complexes
PA	Parthenogenetic activation
DMEM	Dulbecco’s Modified Eagle’s Medium
TCM-199	Tissue culture medium 199
eCG	Equine chorionic gonadotropin
hCG	Human chorionic gonadotropin
PZM-5	Porcine zygote medium-5
